# The effect of bisphosphonate medication on orthodontics and orthognathic surgery in patients with osteogenesis imperfecta

**DOI:** 10.3205/iprs000132

**Published:** 2019-03-29

**Authors:** Reinhard E. Friedrich, Hanna A. Scheuer, Wolf Höltje

**Affiliations:** 1 Department of Oral and Craniomaxillofacial Surgery, Eppendorf University Hospital, University of Hamburg, Hamburg, Germany; 2 Kieferpraxis Lokstedt, Hamburg, Germany; 3 Oral and Craniomaxillofacial Surgery, Klinikum Nord, Hamburg, Germany

**Keywords:** osteogenesis imperfecta, orthodontics, orthognathic surgery, bisphosphonate, oral surgery

## Abstract

**Introduction:** Osteogenesis imperfecta (OI) is a genetic disease that primarily affects bone formation and metabolism. Craniofacial malformations belong to the broad spectrum of the OI phenotype. The introduction of bisphosphonate medications was intended to counteract the significant brittleness of the bones of OI patients. In connection with the application of bisphosphonates, drug-associated osteonecrosis of the jaw has become known as an undesirable effect of the therapeutically intended reduction of osteoclast activity. Originally, the pharmacological inhibition of bone loss was mainly used in oncological therapy. For some time now, osteoporosis has also been treated with substances that inhibit bone resorption.

In OI, malposition of the jaws is relatively common, in particular retrognathia of the maxilla and progeny of the mandible. The literature discloses complications of orthognathic surgery in OI patients. Previous literature reviews suggest that bisphosphonate medication has no significant impact on the performance of and healing after oral surgery in OI patients.

**Material** **and** **methods:** An essential prerequisite of a therapy adapted to the patient’s condition is the knowledge of the patient's medical history. This case report describes the orthodontic-surgical treatment of an OI patient and the treatment experience derived in dealing with the condition. The unusual circumstance of this case is that the patient had concealed both his underlying disease and his medication during the current treatment period. In addition, the relevant literature is evaluated for combining the keywords OI, orthodontic therapy, bisphosphonates, and orthognathic surgery.

**Results:** Based on the literature and our own experience, it is concluded that orthodontic treatment with bisphosphonate medication can also be carried out in OI patients. However, considerably greater forces and longer time intervals should be scheduled for each treatment. Orthognathic surgery with bisphosphonate medication turned out to be uncomplicated in our own case, considering the underlying disease in the planning of surgical procedures. However, there have been very few reports of OI patients in whom orthodontic-surgical treatment of jaw malformation has been performed with bisphosphonate medication.

**Conclusion:** Taking into account the reported experience of severe complications of orthognathic surgery, the multiple documented adjustments to the treatment strategy of OI patients should be taken seriously. The basic condition of therapy adapted to the disease is that the patient informs the practitioner in an appropriate manner about his or her state of health.

## Zusammenfassung

**Einleitung:** Die Osteogenesis imperfecta (OI) ist eine genetische Erkrankung, die vor allem den Knochenaufbau und -stoffwechsel beeinflusst. Kraniofaziale Fehlbildungen gehören zum weiten Spektrum des OI-Phänotyps. Durch die Einführung der Medikation mit Bisphosphonaten wurde intendiert, der oft erheblichen Brüchigkeit der Knochen der OI-Patienten entgegenzuwirken. In Zusammenhang mit der Applikation von Bisphosphonaten ist die Medikamenten-assoziierte Osteonekrose der Kiefer als unerwünschte Wirkung der therapeutischen Verzögerung des Knochenabbaus bekannt geworden, vornehmlich bei Patienten unter onkologischer Medikation, aber auch während der Behandlung der Osteoporose.

Bei OI-Patienten sind Fehlstellungen der Kiefer relativ häufig, insbesondere die progene Stellung der Mandibula in der sagittalen Ebene. Aus der Literatur sind Komplikationen der orthognathischen Chirurgie bei OI-Patienten bekannt. Bisherige Auswertungen der Literatur legen die Schlussfolgerung nahe, dass die Bisphosphonat-Medikation keinen wesentlichen Einfluss auf die Durchführung von und die Heilung nach oralchirurgischen Eingriffen bei OI-Patienten hat.

**Material** **und** **Methoden:** Wesentliche Voraussetzung einer an die Verhältnisse angepassten Therapie ist die Kenntnis über die Vorgeschichte des Patienten. Dieser Fallbericht beschreibt die orthodontisch-chirurgische Behandlung eines OI-Patienten, der über eine längere Zeit seine Knochenerkrankung und Medikation gegenüber den behandelnden Ärzten verschwiegen hat, und die aus der Behandlung abgeleiteten Erfahrungen im Umgang mit dieser Erkrankung. Zusätzlich wird die relevante Literatur zur Kombination der Schlüsselwörter OI, orthodontic therapy (kieferorthopädische Therapie), bisphosphonates (Bisphosphonate) und orthognathic surgery (orthognathische Chirurgie) ausgewertet.

**Ergebnisse:** Aus den Angaben der Literatur und eigenen Erfahrungen wird gefolgert, dass die orthodontische Therapie unter Bisphosphonat-Medikation auch bei OI-Patienten durchgeführt werden kann. Allerdings sollten in die Planung die Notwendigkeit für eine erheblich größere Krafteinwirkung auf die Zähne und längere Zeitintervalle des jeweiligen Behandlungsschrittes einfließen. Die orthognatische Chirurgie unter Bisphosphonat-Medikation gestaltete sich im eigenen Fall unter Beachtung der Grunderkrankung als unkompliziert. Allerdings sind bisher nur sehr wenige Berichte über OI-Patienten bekannt geworden, für die eine orthodontisch-chirurgische Behandlung der Kieferfehlstellung bei Bisphosphonat-Applikation durchgeführt worden ist.

**Schlussfolgerung:** Unter Berücksichtigung der auf Einzelberichten basierenden Erfahrungen mit schweren Komplikationen der orthognatischen Chirurgie sollten die mehrfach dokumentierten Anpassungen der Behandlungsstrategie für OI-Patienten ernst genommen werden. Grundvoraussetzung der an die Erkrankung angepassten Therapie ist die angemessene Information der Patienten über ihren Gesundheitszustand.

## Introduction

Osteogenesis imperfecta (OI) is a collective term for a group of rare diseases that affect the constitution and metabolism of bone [1], [2], [3], [4] and, in many cases, also the teeth [5]. The majority of patients with OI are subject to a disease that complies with the rules of autosomal dominant inheritance [6]. For the majority of cases, mutations coding for collagen I synthesis or affecting collagen I metabolism have been detected [4], [7]. OI is characteristically associated with a high rate of non-traumatic bone fractures [1] (Table 1 (Tab. 1)). The disease is also known as “brittle bone disease” [8]. OI is classified into types according to severity grades and genetic findings [4], [6]. The quality of life of patients with OI correlates with the severity of the disease [9]. Severe forms of OI pose great challenges to the treating physicians and the caregivers of the patients. However, orthopedic surgery in and intensified medical care for OI patients enables many people to have an active life despite repeated outpatient or inpatient treatment of fractures and other OI-related diseases [10], [11]. 

Alterations in the shape of teeth and structure of the hard tissues of teeth have also been demonstrated for a subset of OI patients. This condition is denoted as dentinogenesis imperfecta (DI) (in OI) [5], [12], [13], [14], [15], [16]. Patients with OI may also have further developmental disorders of tissues (e.g., vascular, muscular or ocular symptoms and findings) [1], [6]. The periosteum is also affected in the disease [17]. 

The skull may be affected in different regions by OI [18]. Basilar impressions may require skull base surgical procedures [19]. Maxillary hypoplasia and mandibular prognathism are known parts of the OI phenotype that may cause severe disfiguration [16]. However, severe disorders in relation to the jaw are rarely reported in OI [20]. Cephalometric examinations on OI patients show a normal distribution for most of the measurements in the numerically predominant type I [20].

Malocclusion and dysgnathia associated with OI can be treated by orthodontic and surgical procedures. However, only case reports on orthognathic surgery in OI patients have been published [21], [22], [23], [24], [25], [26], [27], [28], [29], [30], [31], [32], [33], [34], [35], [36], [37], [38], [39], [40].

Bisphosphonates (BPs) have been recommended for the treatment of patients with OI to reduce the risk of fractures [41]. Subsequently, BP medication rapidly has become a standard therapy for patients with OI [42]. BP medication appears to increase bone density in OI patients. However, the question of whether the rate of fracture in patients with OI can be significantly reduced when treated with BP has not yet been conclusively clarified [43], [44]. Other authors see BP medications as a major cause of the reduced risk of bone fractures in OI patients in general [4].

BP medication could be a problem for the planning of procedures in orthodontics and orthognathic surgery [45], because these drugs can affect the orthodontic movement of the teeth [32], [46], [47], [48] as well as the healing of the osteotomized bones [49]. Furthermore, the risk of osteonecrosis with BP medication [40], [50], [51], [52] for this patient group has not been sufficiently assessed [53]. A prerequisite for intensified patient care is the gathering of correct information about pre-exisiting diseases and medical treatments by the treating physicians. In the present case, treatment-relevant information was disclosed by the patient up to shortly before orthodontically prepared orthognathic surgery. This patient was diagnosed with OI and had been on medication for many years. This report is intended to supplement the literature on orthodontic therapy and orthognathic surgery in OI, which is limited to relatively few case-related publications, with special emphasis on the effects of long-term antiresorptive drugs on the procedures.

## Case description

A 24-year-old male patient was referred for orthodontic treatment and orthognathic surgery in order to correct his anomalies of tooth position and jaw relations. According to his statements, previous surgical treatment offers to correct his appearance included tooth correction as well as chin augmentation. These treatment options had been unsuitable in the patient's assessment for adequately implementing his desire to change his profile.

The general medical history of the patient did not indicate any limitation of eventual orthodontic therapy combined with surgery. He expressly denied any serious illness. He reported that his limping walk was due to previous injuries. 

Later, the patient acknowledged that he had deliberately made inaccurate statements about his health condition, because he had previously been refused treatment several times for his tooth anomalies when he had made known his underlying disease and medication.

**Figure 1:**
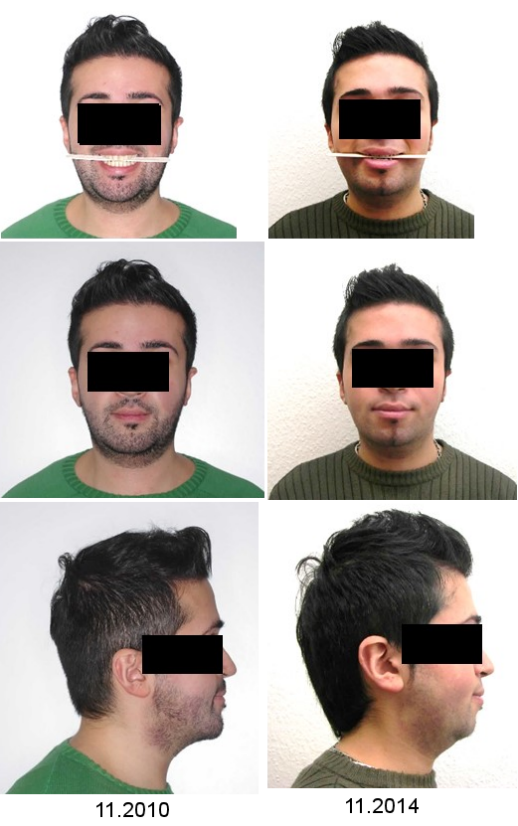
Left column: photographs prior to any therapy, right column: photographs after completion of therapy. En face with wooden spatula between the rows of teeth (top), closed mouth (middle), and from right side (bottom).

**Figure 2:**
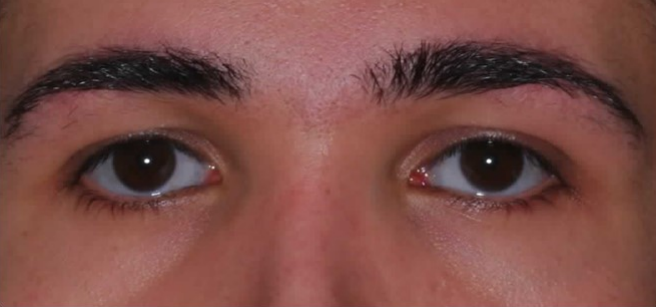
Detail of the patient’s face in en face photograph illustrating slightly bluish sclera

**Figure 3:**
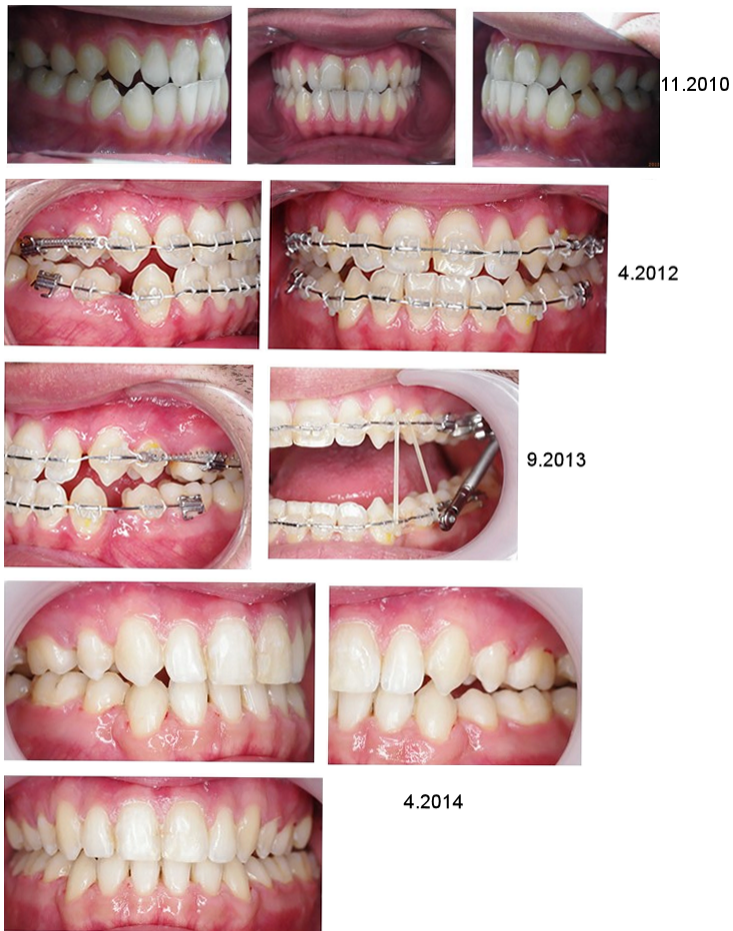
Photographic documentation of the change in position of teeth and jaw during orthodontic-surgical therapy. First series: View of teeth in occlusal contact from the right-front point of view (left), en face (center), and left-front point of view (right) before treatment. Second series: View of the teeth in occlusal contact from the right-front point of view (left) and en face (right) after orthodontic treatment. Third series: The teeth in the left-front point of view in occlusal contact after orthodontic treatment (4/2012) (left) and with inserted Forex spring (right) more than 1 year later. Fourth and fifth series: Final documentation of the tooth position in maximum tooth contact from the right-front point of view (left), the left-front point of view (right), and en face (bottom).

The patient was of relatively short stature with an asymmetrically developed face and maxillary hypoplasia in the sagittal plane (Figure 1 (Fig. 1)). His sclera had a slightly bluish tint, but this was only noticed in connection with the later diagnosis (Figure 2 (Fig. 2)). Intraoral findings at first examination included a class III occlusion (Figure 3 (Fig. 3)). Permanent dentition was complete, including wisdom teeth. The patient's teeth were of normal size and shape. The front teeth had increased translucency upon frontal illumination in the area of the incisal edges and in the proximal areas. There were no attritions of occlusal surfaces that exceeded age-appropriate wear and tear. In conjunction with the later diagnosed OI and respective medical reports on this subject, the dental findings did not indicate any clues for DI.

## Orthodontics

### Cephalometric diagnosis

On attendance, the patient’s facial type was extremely prognathic (SNA=90.3°) with an extraordinarily small skull base angle (NS-Ba=117°). The sagittal interbase relationship was normal (ANB=4.1°). Vertically, the angulation of the mandible was open (ML-NSL=35.8°). The lower front teeth were extremely protruded (UK1-NB=31°, UK1_NB=10.3 mm). In addition, the chin was in retroposition (Pg-NB=–2 mm). The transverse relations of the jaws showed bimaxillary asymmetry to the right with a tilted occlusal plane and deviation of the mandible by 6 mm. The tooth position showed a sagittal class III relation with displacement by half a premolar as well as an anterior crossbite by 2 mm (Table 2 (Tab. 2), definition of abbreviations see Table 3 (Tab. 3)).

In the vertical dimension, overbite was only 0.5 mm combined with open bite in the canine region. In this region, there was also a bilateral transverse crossbite. The upper midline was tilted to the right by 1 mm. There was substantial crowding in the upper and lower jaw. 

### Treatment plan for jaw and teeth relations

Bimaxillary repositioning with advancing genioplasty was planned in order to improve skeletal relations. The extraction of teeth (four premolars, one per quadrant) was necessary for the correction of the mismatch of tooth and jaw size as well as the formation of the dental arch allowing retrusion of the protruded lower front teeth.

### Treatment

The application of brackets on the teeth started with an 0.018 inch system. The brackets were bilaterally fixed in the upper jaw from canine to second molar and in the lower jaw from canine to first molar. Following this procedure, the extraction of teeth was carried out. The retraction of the canines started with 0.016 x 0.016 inch Nitinol™ arches and elastic chains. As the movement of teeth seemed to be unusually slow, the power was raised three months later to 0.016 x 0.016 inch stainless steel with red tension springs. After 7 months of treatment, the front teeth were included in the appliance. The tension springs were used for 8 months for retraction of the front teeth. This distribution of force was maintained until surgery.

After the second surgical procedure and removal of maxilla-mandibular fixation (MMF), orthodontic treatment consisted of intermaxillary elastics in class II direction connecting the upper lateral front tooth and canine to the first lower molar on both sides. The BP medication was restarted, as directed by the attending orthopedist. Two months after the surgical procedures, the attempts to close the extraction gaps went on: for one month with red tension springs and one month with elastics from the second lower front tooth to the first lower molar on the same side, renewed twice a day. As there was no improvement in tooth movement speed, springs were used again. 

Six months after surgery, the patient got really bored with the orthodontic appliance, but gaps between teeth still were not closed. Therefore, intermaxillary springs in class II direction (Forsus™) were installed for an unusually long time (9 months). This appliance is extremely effective and is usually applied for 3 to 5 months in class II cases to correct a whole premolar width. After this procedure, the intended gap closure was continued with elastic chains. Six months later the fixed appliance was removed, even though the gaps were not closed completely. The patient got fixed retainers in the upper and lower front and had to wear removable appliances with screws to close the gaps and a class II component. That means the orthodontic appliance was in place for a total of 3 years, with 1 1/2 years of this time being after surgery. 

Compared to an estimated treatment time for the formation of the dental arches and correction of a malocclusion without the mentioned conditions, the orthodontic therapy presented in our case was prolonged by approximately two to three times. 

**Figure 4:**
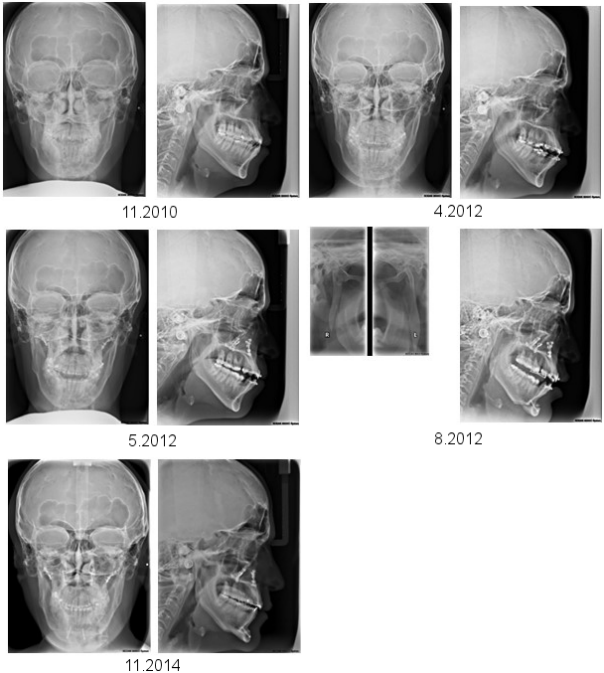
A series of cephalometric radiographs depicting the patient in anterior-posterior (left) or lateral projection (except the fourth series of radiographs). The first series shows the dental and skeletal relations prior to treatment. The second series shows the final position after completion of orthodontics prior to the first surgical procedure. The third series shows the position of the bones following maxillary osteotomy. The fourth series shows the vertical osteotomy of each mandibular ramus (left side), and the position of the jaws, including chin osteotomy, after the second surgical procedure. Finally, the fifth series demonstrates the relation of the jaws after completion of post-surgical orthodontics.

**Figure 5:**
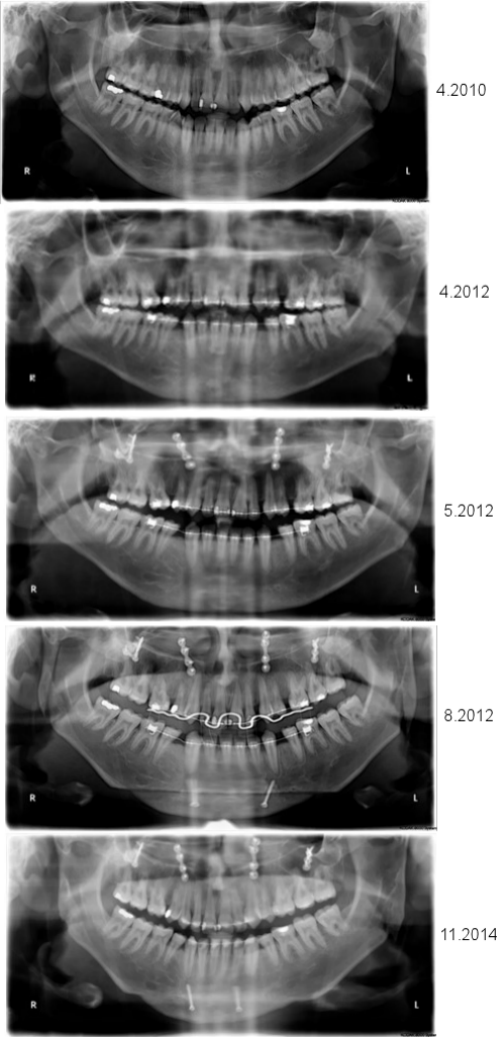
A series of orthopantomograms depicting the patient’s jaw during orthodontic-surgical treatment. From top to bottom: 1. situation prior to any therapy; 2. after orthodontics; 3. after maxillary osteotomy; 4. after mandibular osteotomies; and 5. after completion of post-surgical orthodontics.

The orthodontic treatment documentation is shown in Figure 1 (Fig. 1), Figure 3 (Fig. 3), Figure 4 (Fig. 4), Figure 5 (Fig. 5), and Table 2 (Tab. 2).

### Antiresorptive drugs medication

The unusually large forces that had to be applied to the tooth movement had increased the attention to the patient. After completion of pre-surgical orthodontics and during preparation for the surgical procedure, deformities of the forearm bones were noticeable due to summer clothing. Apparently, this finding was the result of a fracture. In combination with the obvious asymmetry of the legs, the question was repeated to the patient regarding an existing bone disease. This time the patient admitted to having suffered from a generalized childhood bone disorder that had been classified as OI.

After the patient admitted to having concealed a serious bone disease from his treating physicians, so as not to jeopardize the execution of his desired orthodontic-surgical treatment, several medical treatment documents could be requested, from which a complex, long-term illness could be traced. The patient had already been diagnosed with OI as a 14-year-old. Since the sixth month of life, he had experienced about 20 fractures until the establishment of the pediatric diagnosis. The medical documents showed that he had already been classified as OI type I due to his medical history, proof of blue sclera, thoracic kyphosis, and DI. The diagnosis was made simultaneously with his 18-year-old brother, who also showed DI but was less severely affected as far as the number of bone fractures is concerned. However, after the diagnosis, a BP medication was not immediately started. At 21 years of age, the patient received more than 1 year of intravenous (IV) BP therapy with Aredia™ (pamidronate, 30 mg) every 3 months for the prophylaxis of further fractures. Thereafter, the medication was changed to weekly Alendron™ (alendronate, 70 mg) for 1 year. From the age of 24 to 29 years, the patient received IV Bonviva™ (ibandronate, 150 mg, once a month). Indeed, the patient discontinued his current BP medication only for a short period of time after the completion of pre-surgical orthodontics. That means, this measure (“drug holiday”) was effective only during the short period of consecutive orthognathic surgical procedures. After completion of surgery, the patient restarted his antiresorptive medication after consultation with the treating orthopedist. Since the age of 29 years, the patient has been treated with IV Prolia™ (denosumab, 60 mg) every 6 months. 

### Oral surgery

The extractions of four premolars and consecutive wound healing proceeded inconspicuously according to the stage. 

### Orthognathic surgery

The very late knowledge about the patient's bone disease and his current medication led to the decision to control the operative procedure and, therefore, to proceed in individual steps. In our opinion, there was an increased risk of bleeding during and after the procedure. In addition, given the long-term use of BP medication, the capacity for bone healing was not reliable. For this reason, a two-stage procedure was performed in the osteotomies of the jaws. After coordination with his orthopedic doctor, he was told to interrupt the medication.

Surgical therapy took place about 1 year after the beginning of orthodontic therapy (patient age: 26 years). For the surgical correction of jaw positions, an osteotomy was performed at the Le Fort I level with a saw and chisel. After a downfracture of the detached maxilla was performed, the maxilla was mobilized and posteriorly impacted, and the occlusal splint was incorporated. Osteosynthesis of the segment was done with four miniplates. The displacement of the maxilla caused it to rotate posteriorly, which improved the overbite. By correcting the tilted occlusal plane, a unilateral open bite resulted on the left side. For postoperative stabilization, a composite buildup was installed on the first and second molar of the lower left side and was left in place until the second surgical step. The anterior crossbite remained and was corrected by the following mandibular procedure. 

Vertical oblique osteotomy of the mandibular ramus combined with advancing genioplasty was performed 3 1/2 months after the maxillary procedure. This procedure resulted in setback of the mandible and shortening of the ramus on the left side. The right side only received rotation of the ramus with minimal setback. The following advancement of the chin was symmetrical, because the correction for panfacial asymmetry was already performed by jaw surgery. After the mandibular osteotomy, the stabilization resulted from MMF via the orthodontic appliances and integrated splint. The second splint had three perforations, each 1 cm² in size, for easier breathing and allowing liquid nutrition. This second procedure also proceeded without major bleeding. In this case, the intraoperative procedure gave the impression that, without the knowledge of the underlying disease, the operative course of the procedure would not have differed from that of a patient not suffering from OI [32].

The surgical treatment documentation is shown in Figure 1 (Fig. 1), Figure 3 (Fig. 3), Figure 4 (Fig. 4), Figure 5 (Fig. 5), and Table 2 (Tab. 2).

### Histology

Tissue samples of the ablated maxilla in the osteotomy line showed normal bone formation in the histological examination.

### Follow-up

The last follow-up of the patient took place 3 years after the second surgical procedure. At this time, the patient had a stable class I occlusion, no signs of osteonecrosis, and radiologically unremarkable healed osteotomies of the jaw. The patient reported that he had suffered further fractures of the extremities after completion of orthognathic surgery and orthodontic procedures while continuing to receive denosumab medication. The skeletal function of the jaw had remained stable and uncomplicated since the orthodontic-surgical measures.

### Review of the literature

The literature review (Table 4 (Tab. 4)) included 27 reports of jaw osteotomies in patients with OI, including the present case (mean age: 22.59 years). Thirteen women and 12 men were treated (no information: two patients). The age of the women at the time of treatment was on average 23.38 years (range: 17.5 to 36 years) and that of men was on average 22.5 years (range: 12 to 40 years). 

The diagnosis of OI was certain in all cases (Table 1 (Tab. 1)). Typing of OI was missing for seven patients. In another case, the typing could not unambiguously distinguish between two classes. OI type I was the predominant diagnosis (14 patients), followed by a few cases that had either OI type III (three patients) or type IV (two patients). None of the reports on orthognathic surgery in OI patients refer to an OI severity grading system when describing the individual phenotype of the respective patient(s). However, orofacial or maxillofacial findings are not an assessment factor for a recently proposed severity classification [4].

A Le Fort I osteotomy was chosen as access to the skull base solely in order to treat basilar impression in a report detailing treatment of four patients. The report mentioned some maxillary advancement combined with spine surgery in one of these cases. Transposition of the divided jaws was carried out in all other cases or at least intended.

In 13 cases, only one jaw was osteotomized (women: 4, men: 9). Both jaws were osteotomized in 14 cases, of which two were temporally separate procedures for the same respective jaw. 

Osteotomies at Le Fort I level were made in 21 patients (women: 11, men 8, unspecified: 2). Lower jaw osteotomies (including genioplasty) were performed in 20 patients (women: 8, men: 10, unspecified: 2). Maxillary osteotomies were performed in all to achieve sagittal advancement of the maxilla.

In the lower jaw, the osteotomy aimed to relocate the corpus. Sagittal osteotomy of the mandibular ramus was the most frequently used technique (10 cases), followed by vertical ramus osteotomy (5 cases) and osteotomy within the mandibular corpus (3 cases). From the reports, it can be derived that the osteotomy of the lower jaw was used for setback of the corpus.

The osteosynthesis material corresponds to the developments of the procedures over the long period in which the recorded reports have been carried out. Wire osteosynthesis, which was previously used, has been replaced almost everywhere in later operations by miniplate osteosynthesis. However, a recent report shows that occasionally wire osteosynthesis is still used, combined with screw osteosynthesis [31]. While some reports highlight the fragility and narrowness of bones, none of the reports on OI patients subjected to orthognathic procedures appear to have another fracture outside the selected osteotomy line that would have required surgical care [39].

BP medications are mentioned in three reports and are listed in detail for our own case. All other reports mention no such medication. For OI patients whose osteotomies were performed before the year 2000, it is assumed that BP medication is unlikely. In one case, BP medication was only mentioned and not specified. In another case, monotherapy with alendronate was performed; and in our own case, several medications of this type and another drug with similar activity were used before and after treatment. A short phase of interruption of medication for the operative treatment phase was known for two of the three cases. The fourth report was about successful orthognathic surgery on an OI patient. In this case, only a small amount of treatment information was found in the brief communication [38].

OI diagnosis had an impact on planning of the surgical strategy for a variety of reasons. The timing of the diagnosis had also been an important factor in therapy decisions. In one case, referring to the increased risk of complications during osteotomy, the surgical concept was adapted whereby the incomplete dentition allowed for prosthetic compensation of the unbalanced jaw relation [21]. In another case, distraction of the segment was performed in the upper jaw after osteotomy instead of immediate transposition for the same reasons, as to avoid bleeding complications [33]. In a third case, the bimaxillary operation was divided into two separate procedures, one jaw osteotomized each to minimize blood loss during and after surgery. In this case, for religious reasons, the patient had already refused transfusion of blood prior to surgical treatment [30]. Despite these precautions with a long interval between interventions (7 months), perioperative hemorrhage occurred at the second procedure, necessitating omission of bone grafting [30]. In our own case, the diagnosis of OI combined with the knowledge of pharmacotherapy led to the decision to osteotomize each jaw individually in one operation and to wait for a longer period of time between the procedures. These two patients are the only bimaxillary osteotomies in this review that were planned as two-time interventions. Another report indicates that previously unknown OI was noticed in two cases by the surgeon during osteotomy of the jaw due to significant brittleness of the bones [39]. This finding has led to the adaptation of orthognathic intervention planning. In both cases, the osteotomy of the second jaw was omitted. Intraoperative suspicion of OI or very late knowledge of the disease is therefore available for at least three out of 27 patients.

Surgical complications, such as severe bleeding from the surgical situs, have been reported only for patients in whom no administration of BP medication was known or suspected. These reports described incidents more than 20 years ago. The brittleness of the bones and the difficult osteotomy are emphasized in individual reports.

## Discussion

This report demonstrates the successful orthodontic-surgical treatment of a patient with OI who had been on different BP medications several years prior to the current procedures and had concealed his medical history and current drug intake from his present physicians during relevant treatment phases on teeth and bones.

This report shows that the reinforcement of orthodontic appliances for tooth movement is clearly required in patients with pharmacological reduction of bone resorption. The surgical measures were adjusted to the late knowledge of the underlying disease and current drug use to address potentially impaired bone healing and the risk of major bleeding during surgery. Therefore, the jaws were individually osteotomized. Between the surgical appointments, the uncomplicated healing of the first jaw was awaited.

The report also shows that the procedures used have provided stable skeletal and dentoalveolar conditions over a reasonable follow-up period. In this case, the antiresorptive drugs had a significant influence on the orthodontic treatment regarding the equipment and forces to be used for the mobilization of the teeth and treatment time. However, the stability of the final orthodontic tooth setting was maintained at the follow-up intervals. Likewise, after completion of therapy, no bone necrosis or periodontal disease was observed during long-term administration of different drugs that have an antiresorptive effect on bone metabolism.

### Clinics and morphology

#### Clinics in OI

OI is an umbrella term to classify inherited diseases that are caused by a plethora of mutations and consecutive disorders of protein synthesis and metabolism, which mainly leads to defective collagen I function [4]. OI is a rarely diagnosed disease affecting about 1 in 5,000 to 10,000 individuals [4]. The repeatedly revised classification of OI is based on the assessment of phenotype and determination of genotype [4]. At present, mainly four [16], [54] or five [4] types are distinguished. However, much more subtypes are classified in clinical research through improved genetic analyses of affected individuals [4], [16]. Nevertheless, the assessment of clinical severity of the disease is far more important for the classification of OI and the initiation of a care plan than the identification of genotype [4]. 

Phenotype varies considerably between the types and even amongst family members [4]. OI type I is estimated to account for about 60% of cases [55]. Obviously, the severity of OI phenotype varies with the quality of collagen I protein [3], [56], [57]. Whereas decreased degradation of collagen I is associated with a relatively mild phenotype, abnormal collagen I production causes more severe manifestation of OI [57]. Both the incidence of fractures during birth and the number of fractures are not rated as reliable indicators of the severity of the disease [2]. The spectrum of general findings is wide-ranging in relation to the patients who received orthognathic surgery (Table 4 (Tab. 4)).

#### Craniofacial morphology in OI

In OI type I, all craniofacial bones tend to be smaller than normal [25]. This assessment has been made more precise by cephalometric examinations by Jensen and Lund [3], who demonstrated reduced mandibular length and posterior height compared to a control group for all types of OI. In addition, the jaw relations in the sagittal and vertical planes were normal in all OI patients (except female OI type III patients). The anterior facial height was normal in OI type I patients [3]. However, the width of the maxilla was reduced in all OI groups. These examinations were performed on 35 OI type I patients [3]. Others summarize the craniofacial morphology of OI type I patients as “almost normal craniofacial development... with a tendency for a slight decrease in the size of the jaws but still within normal variation” [5]. In contrast, numerous abnormal craniofacial findings are detected in OI type III and IV patients [5]. A reduction in the external shape of hard tissue in OI patients can also affect the teeth. This proportional reduction in the volume of permanent teeth can give the impression that the OI patient has persistent deciduous teeth [5].

In OI type 1, smaller than normal linear measurements were noted upon further cephalometric study [20]. These patients seemed to have a general growth deficit. However, no remarkable craniofacial deformity was registered in this group of OI patients [20]. The authors deduced from their calculations that vertical underdevelopment of the dentoalveolar structures and the condylar process were the main reasons for the relative mandibular prognathism in some patients with OI. However, no continuous class III skeletal pattern was detected for OI type I patients. It was noticeable in this patient group that both jaws were shorter in the sagittal plane than in the control group. In contrast to previous reports that demonstrated hypoplasia of the maxilla combined with normal or overdeveloped mandibular growth [12], [13], [15], a harmonious class I relationship of the jaws was demonstrated in this group of OI type I patients [20]. However, this statement is mainly based on a historical comparison of cephalometric measurements in OI type I patients with patients from other publications who had developed OI type III or IV [15]. The authors speculated that facial growth impairment will probably remain a characteristic for many OI patients regardless of the widespread use of BP medication. This statement was presented without elaborating on the severity of craniofacial findings in relation to the type of OI [20]. Recently, Arponen et al. [58] confirmed this statement after observing the development of cranial base pathology in OI patients regardless of BP therapy. The authors speculated that administration of BPs early in life may delay the development of craniocervical junction pathology. 

### Orthodontics

#### Orthodontics in patients taking BP medication

Early on, the therapeutic use of BP medication was considered to contribute to difficulty in the orthodontic movement of teeth [45]. Orthodontic treatment of patients on BP medication has been critically assessed in several reviews [47], [59], [60]. There is limited clinical data on the influence of BPs on orthodontic therapy in patients on which recommendations can be based [46], [59], [61], [62], [63], [64], [65], [66], [67]. In general, it is pointed out that although orthodontic treatment can be successfully performed with respect to largely atraumatic procedures, treatment time can sometimes be considerably prolonged, the amount of force applied for tooth movement must be increased, and the goal of tooth movement may not be achieved [47], [59], [60]. 

A retrospective clinical study has analyzed the effect of BPs as a risk factor of adult orthodontic therapy. Inclusion criteria were women over the age of 50 years. This group was selected because these patients were considered at risk for the development of osteoporosis and, therefore, a target group of non-oncological BP therapy [46]. Based on the data of a group of 20 female patients taking BP medication and receiving orthodontic therapy, these authors identified significantly prolonged treatment times following tooth extractions in the course of orthodontic treatment. However, no osteonecrosis was noted in these cases. Incomplete space closure and poor root parallelism were significantly elevated risks for BP-treated patients.

Krieger et al. [66] reported about a patient who had been planned for full-mouth rehabilitation, including orthodontics. The patient’s dentition was reduced to anterior teeth in both jaws. Dental implants were placed in order to extend the dental arch. Unexpectedly, the authors found out that the patient’s general physician had introduced (and not communicated) an oral BP medication for the treatment of osteoporosis. It became evident that the orthodontic therapy was carried out for about 6 months in parallel to the BP medication. Orthodontic therapy was successfully performed with maximum anchorage to the previously inserted implants. The increased tooth mobility at the end of the therapy was not estimated as a consequence of BP medication, because there was considerable periodontal disease. This case is similar to our own report regarding the lack of information on essential medication at the time of treatment. In the same way, our patient continued to take BP medication during orthodontic therapy [59]. On the other hand, Krieger et al. [66] reported a lower BP medication dose (alendronate, 70 mg/week) over a relatively short period of time, whereas our patient had been treated with both IV and oral BPs for a long time prior to orthodontic tooth movement. These differences in drug treatment can also serve to explain why, in contrast to Krieger et al.'s case, the orthodontic therapy of our patient had required considerably higher forces. Zahrowski [59] had already emphasized that patients had failed to report BP medication to the orthodontist, because they had considered this medication irrelevant to the dental treatment.

After reporting their case with OI treatment, Krieger et al. [47] reviewed orthodontic therapy in nine patients with a history of BP medication, including their own case (n=9). Medical treatment was unknown to the physician in an additional case. In this additional case, several findings were registered: hypermineralization at the extraction site of teeth, sclerotic bone, widened periodontal gaps, decelerated tooth movement, and continuation of side effects despite stopping medication [59]. Most patients (n=6) in the review article had been treated with BP medication for osteoporosis, but generalized bone disease (fibrous dysplasia) [64], metabolic diseases (Addison’s disease), and malignancy with bone manifestations were also among the indications for drug-induced inhibition of osteoclasts [62].

Following autologous bone transplantation, orthodontic tooth movement was also successfully performed in a patient with long-standing oral BP drug therapy for osteoporosis [63]. Additionally, orthodontic appliances were successfully used to perform forced eruption of destroyed teeth to assist with tooth extraction in a patient with a long-standing history of BP medication for osteoporosis [65]. A further report emphasized the capability of orthodontic tooth movement in a patient with a history of both local irradiation therapy and BP medication for malignant lymphoma [67].

However, the vast majority of studies on the influence of BP medication on intended tooth movement is based on animal experiments [68]. Some of these publications show delayed and less extensive movement of teeth in BP-treated animals under the influence of orthodontic forces. The relapse after exposure to the forces is also lower than in the control groups without BP medication. This assessment applies equally to experimental maxillary expansion and mandibular distraction. However, other studies reported less root resorption and faster movement of teeth during orthodontics in experimental animals [68]. As a result, the experimental data regarding the effect of BP medications on teeth undergoing orthodontic procedures, at present, has to be assessed as ambiguous [47]. 

#### Orthodontics in OI

Some reports detail experiences in orthodontic treatment of selected individuals suffering from OI [32], [69] or report on general experiences in orthodontic treatment of this group of patients [5], [32]. The indications and conditions for orthodontic treatment are different for each diagnostic OI group. Although class III malocclusion is known in OI type I, III, and IV, the frequency and severity of malocclusion varies significantly between these groups. Class III malocclusion is usually much milder in OI type I and can be treated in private orthodontic practice [5]. The closure of the open bite by orthodontic measures is significantly more difficult or impossible to achieve in some OI patients [5]. The posterior open bite occurs predominantly in OI type III and IV patients and independently of BP therapy [5].

Orthodontic therapy is an integral part of the vast majority of OI patients where orthognathic surgery was performed, usually for decompensation of tooth positions (Table 4 (Tab. 4)). These orthodontic treatments, as part of a surgical treatment concept, were tailored to the individual case, are heterogeneous in their measures, and documented in very different quality. It should be noted, however, that the conclusion of Hartsfield et al. [32] still is valid and there are still no published clinical prospective studies regarding the performance of orthodontic therapy in OI patients. Indeed, the majority of orthodontic reports on OI patients are diagnostic [13], [20], [58], [48], [70], [71].

Class III malocclusion was found in 18 (66%) of 28 OI patients [13], predominantly in those with a family history of this condition. However, class III malocclusion was also noted in OI type III and in a single OI type IV patient. The authors point out that at the time of their examination, the incidence of this condition in the general population was 3% to 8%. The authors rate the greater frequency of class III malocclusion in OI type III as a consequence of the more pronounced involvement of the skeleton, which is characteristic for this patient group. In 16 out of 18 patients, a posterior unilateral or bilateral crossbite was detected, and crowding of the teeth was nominal or absent. Another finding was the higher rate of tooth impactions in OI patients. This report did not detail any information about orthodontic therapy in these patients [13].

A recent report confirms the high rate of class III malocclusion in a large Canadian study group of OI patients [70]. The aim of the study was to collect orthodontic records prior to a potential orthodontic treatment. The distribution of diagnostic groups deviated significantly from the frequency distribution of OI in the population. Predominantly affected were patients with OI type III, IV, and V (84%). Relative mandibular dentoalveolar prognathism was present in the majority of the patients. All patients had received or were still receiving BP therapy. The authors agreed with earlier explanations [15] of the pathological relation of the jaws in OI patients, based on relative prognathism as the result of obstructed anterior-inferior maxilla movement [3]. The inhibition of jaw movement is caused by a primary growth defect at the cranial base combined with vertical underdevelopment of the dentoalveolar structures and condylar process [70].

A recent study from Vietnam on the need for orthodontic therapy in OI confirms the high prevalence of class III malocclusion in a large population of patients. The frequency distribution confirms the well-known numerical superiority of type I in OI patients [71]. OI correlated significantly with reverse overjet and missing teeth. In contrast to the Canadian study patients, most of the Vietnamese OI patients had not yet received BP medication.

In two young patients with OI the Fränkel appliance was successfully used to prohibit anterior crossbite in permanent dentition and to improve facial profile [72].

#### Orthodontics in OI and BP medication

There are very few reports of the influence of BP medication on orthodontic therapy in OI patients. Abukabbos and Al-Sineedi [73] used space maintainers to stabilize the position of restored teeth. Obviously, BP medication had no effect on the orthodontic treatment. Kamoun-Goldrat [74] identified delayed tooth eruption in children with OI taking BPs. This delay in tooth eruption in OI patients due to BP medication was distinguishable from the delay in tooth eruption in OI patients without this drug. In a further case, complex orthodontic measures were performed to assist with tooth eruption, to correct overbite and crossbite, and to close spaces. The treatment lasted almost 3 years and included surgical procedures as well as keeping in the row of teeth for later prosthetic measures [32]. In a recent abstract, the orthodontic partial shaping of the dental arch is described in connection with the complication-free extraction of premolars and wisdom teeth in an OI patient taking BP medication (neridronate) [75].

### Surgery

#### OI and orthopedic surgery of the axial skeleton

Orthopedic surgery in OI patients enables many people to have an active life despite repeated outpatient or inpatient treatment of fractures [76], [77], [78]. However, bones of adult OI patients appeared to take longer to heal than skeletally healthy individuals [10]. 

#### OI and orthopedic surgery while taking BP medication

An early report detailed that pamidronate therapy was associated with delayed healing of osteotomy sites after intramedullary rodding procedures on long bones in OI patients. Interestingly, the effect of pamidronate on fracture healing in this patient group was not validated after taking into account the effect of age [49]. The previously reported delayed wound healing after osteotomy in patients with OI and BP medication can apparently be accelerated by changing the drug and the surgical technique [78].

#### OI and the risks for osteonecrosis of the jaw and oral surgery while taking BP medication

There is apparently no association between BP medication and osteonecrosis of the jaws in patients with OI [53]. Typical oral surgery procedures (e.g., extraction, osteotomy of impacted teeth, and exposure of retained teeth) and other dental treatments (e.g., pulpectomy and abscess drainage) did not result in osteonecrosis of the jaws in children and adolescents with OI [22], [79], [80], [81], [82], [83], [84], [85], [86], [87]. Individual reports on the insertion of implants in OI patients prove that the osseointegration of these foreign bodies succeeds [33], [40].

Atraumatic techniques should find applications that are not different from those used in patients on BP medication without this genetic background [88]. The risk of BP-associated osteonecrosis of the jaws appears to be significantly increased for adult patients with malignant disease and symptomatic disorders of bone metabolism unrelated to OI [89]. There is at least one report that described jaw osteonecrosis during orthodontic treatment where the oncological patient needed surgical advice [62]. Nevertheless, it was stressed that systematic studies on the topic of the risk of jaw osteonecrosis in OI patients taking BP drugs still are pending [53].

#### OI and the risk for jaw fractures

Fractures of the jaws are rarely reported for OI. In individual cases, the jaw fracture can be pioneering for the diagnosis [90]. Tooth extractions can lead to mandibular fractures in individual cases [91]. Lower jaw fractures in OI patients were also observed without adequate external cause and were considered spontaneous fractures [92], [93]. Mandibular fractures were also seen in children with OI [31], [94]. Reports on the surgical treatment of jaw fractures in OI patients usually describe regular bone healing after osteosynthesis [90], [92], [95], [96], [97]. However, osteosynthesis of a mandibular fracture may be risky in patients with OI, because another fracture may occur during osteosynthesis of the primary fracture [98]. As a rule, miniplates are used for osteosynthesis, but wire osteosynthesis has also been used successfully to fix fractures [99]. As an alternative, the conservative treatment of mandibular fractures has also been used successfully [94], [100]. At least two of the listed OI patients who experienced a mandibular fracture had received BP therapy [92], [97].

An older report describes possible traumatic damage to the facial skull in a larger group of OI patients, but the information remains inaccurate [101]. The report is considered because of the unusually high number of (suspected) trauma cases.

In a review of otological and maxillofacial aspects of OI, Bergstrom [101] describes a group of patients (n=48), some of them had a history of facial skull trauma. Fracture of facial bones was known or likely, at least, in some of these patients. One patient (“OI tarda”) simultaneously had suffered a midfacial bone fracture (“Le Fort II” fracture) and mandibular fracture in a car accident. The consequences of the accident after treatment (“reduction and fixation”, not further specified) are given as asymmetry of the malar region and class III malocclusion. Although the course of treatment is not elaborated, it can be assumed that the treatment measures led to stable ossification of the fragments. This is the only patient in this survey who had fractures in both jaws simultaneously. A second OI tarda patient, who had been involved in a car accident, suffered a nasal fracture. Additionally, five cases had features that were suggestive for the possibility of old or recent fractures. However, no adequate investigations were described that would justify this presumption beyond appearance. Further information on therapy and the course of the trauma patients cannot be found in the report. In particular, there is no comparison to the skeletal condition before the trauma.

#### Orthognathic surgery in OI

Reports of the orthognathic surgical treatment of patients with OI are based on single or multiple case reports (Table 4 (Tab. 4)). However, there are also, so far, only a few reviews of orthognathic surgery in patients taking BP medication without this genetic background [102]. The evaluation of orthognathic surgical procedures (including distraction) shows stable results in the majority of procedures. Complications are rarely experienced, although difficult to master in individual cases (see below). In addition to the en-bloc transposition of jaw segments in order to correct jaw relations, other osteotomies and augmentation procedures of the jaws were performed in OI patients.

- Maxillotomy at the Le Fort I level with sagittal splitting of the maxilla was successful and complication-free. In patients with OI, it was used as a surgical approach to the skull base to treat basilar invagination [19]. These cases have been taken into account in the evaluation of the literature.
- Rapid maxillary expansion under IV BP therapy was successfully performed in three cases [103]. BP therapy was discontinued during the active phase of palatal expansion. The regular ossification of the expansion gap was radiographically documented 1 year after completion of the treatment.
- Le Fort I osteotomy combined with distraction osteogenesis was also used in OI (“late form”), with severe maxillary deficiency and extreme atrophy of the almost toothless alveolar ridge [33]. The procedure was chosen with respect to dental status and in order to reduce the risk for bleeding. Successful advancement proved stable results after the skeletal procedure and integration of cover dentures during an observation period of 4 years (Table 4 (Tab. 4)).
- A single report describes successful bone transport osteogenesis after continuity resection of the mandible in an OI patient [104].

#### Orthognathic surgery in OI patients taking BP medication

Reports of orthognathic surgery in OI patients taking BP medication are rare so far. Rosén et al. detailed surgical therapy in a patient who had been treated with alendronic acid [37]. Surgical procedures were successfully performed, accompanied by 850 mL blood loss during surgery and with some bleeds during the recovery periods that needed no intervention. Orthodontics were performed prior to and after surgery. It is not reported, however, if the known medication had an effect on this part of the treatment. The second report is only an abstract that details successful surgical procedures in an OI patient with a history of BP therapy [38]. The third report is the published illustration of an oral presentation about orthognathic surgery of an OI patient taking BP medication [40].

#### Treatment results and stability of surgical and combined surgical-orthodontic procedures

Some authors do not discuss the treatment success, apparently because:

1. treatment had been continued prosthetically [21];
2. diagnosis and treatment of complications is in the foreground of the intention of the report [22], [23];
3. report mainly deals with non-surgical measures during surgical treatment [27] or;
4. report is focused on the treatment of OI patients with craniofacial malformations in general [26].

Further reports only give a brief description of the treatment results, which do not allow further conclusions about the entire treatment [38], [40].

Statements on the success of the treatment are generally qualitative and focus on occlusion and facial appearance. It is reported that the treatment led to a stable result of the skeletal and/or dental position, and the esthetic result was satisfactory or good [22], [25], [29], [36], [37], [38], [39]. At least, the relevant parts of the maxillomandibular relationships maintained stable positions [30]. In one case, severe complications occurred during and after the surgical procedure that required a repeated intervention in the surgical site [22]. Nevertheless, the planned positions of the jaws remained stable 30 years after the procedure [36].

In one case, noticeable occlusal relapse was noted and explained by patient noncompliance [39]. In another case, the posterior crossbite returned postoperatively [30]. A further case required orthodontic therapy for relapse of anterior open bite and long-term retention therapy [25].

Transmaxillary access to the spine for the treatment of basilar impressions in OI patients has been associated with some complications (e.g., difficulty in swallowing and fistulas) [19] that have not been reported for Le Fort I osteotomies in this form or at all in orthognathic osteotomies in this group of patients. In fact, these interventions primarily had not been done for permanent transposition of the upper jaw. Nevertheless, satisfactory functional results have finally been registered in these cases as well.

In summary, the individual reports show that combined orthodontic-surgical therapy of OI patients with severe malocclusion leads to satisfactory esthetic and functional results that have been controlled in several cases over many years.

#### Complications during orthognathic surgery in OI patients

The majority of surgical procedures were carried out without complications, neither during the procedure nor during follow-up (Table 4 (Tab. 4)). Only some reports detailed quantitative data on blood loss (mL) [22], [27], [28], [37], [39] or blood/plasma transfusions (mL or units) [23], [24]. Two reports detailed significant and life-threatening bleeding events during orthognathic surgical treatment, which required invasive measures to manage the situation [22], [24], including ligature of the external carotid artery in one case [22], [36]. In another case, severe perioperative hemorrhage occurred during the second orthognathic procedure [30]. Some reports do not mention (abnormal) bleeding [21], [26], [33], [38], the surgical concept was limited to the osteotomy of a jaw to avoid bleeding complications [21], [33], the procedure explicitly was performed without exceptionally increased blood loss [29], [34], give only inaccurate information about blood loss during surgery [30], [32], or the volume loss is detailed to be less than or around 300 mL [27], [37] or up to 1,000 mL at maximum [28], [37].

Noteworthy is a report in which the unusual properties of the bone during the osteotomy of the jaw had led to the suspicion of previously undiagnosed OI. Tashima et al. [39] report on their experience in the treatment of two patients whose physical examination had not suggested a bone disease. The authors had planned a bimaxillary procedure in each patient. The most striking intraoperative signs had been the unusual brittleness of the osteotomized, very thin bones and inadvertent fractures. In both cases, the intraoperative findings during osteotomy of the first jaw resulted in a change in the surgical treatment concept. The intervention in the second, not yet osteotomized, jaw was omitted, and it underwent orthodontic therapy adapted to the new conditions. Wound healing progressed normally and orthodontic treatment in these patients was reported to have been without complications [39].

In one case, revision of the osteotomy sites was considered necessary in order to stabilize the segment positions with wire fixation [31].

#### Recommendations for oral and maxillofacial procedures in the treatment of OI patients

There are general comments on the treatment of OI patients, which advise the practitioner to proceed cautiously and as atraumatic as possible during oral and maxillofacial interventions [32], [59]. These recommendations generally apply to treatments such as dental extractions with no particular reference to the specific type of OI patients. From the review of the literature, it can be concluded that on the one hand complications of oral surgery can occur [91], [94], but on the other hand the number of published complications is relatively low. 

In orthognathic surgical interventions serious complications have occurred in individual cases [22], [24], [31]. However, the vast majority of surgical procedures were carried out without major complications (Table 4 (Tab. 4)), provided that the treating physicians were informed about the disease (Table 1 (Tab. 1)) and the treatment could be tailored to the particular conditions of the patient [39]. 

The recorded orthognathic surgical procedures do not identify any specific type of OI patients selected for this procedure. BP medication had no complications in three proven cases of orthognathic surgery in OI patients.

The known complications from the clinical application of BPs in severely affected individuals have led to formal recommendations for the management of minor and major surgical procedures in the oral and maxillofacial regions in patients taking BP medication [105], [106]. Orthodontists know that tooth movement in OI patients on BP medications can be difficult or even impossible, as shown in clinical work [32] and from experimental studies performed in vitro or in animals [35], [38]. Recommendations for the orthodontic treatment of OI patients on BP medication merely indicate the aggravated treatment conditions and the increased risk of not achieving the intended goal of tooth movement [46]. Orthodontic treatment to prepare for surgery in OI patients has been mentioned in some reports. The orthodontic treatment following the transposition of jaws is also recorded only for some reports (Table 4 (Tab. 4)). Only occasionally, the duration of postoperative orthodontic therapy is mentioned [28], [37]. The descriptions of the measures are relatively unspecific and essentially limited to the postoperative incorporation of elastics, particularly for the treatment of recurrent anterior open bite (Table 4 (Tab. 4)). However, these reports are mainly based on treatment of patients not taking BP medication. As a result, no orthodontic recommendations can be derived in patients with impaired collagen I synthesis and metabolism subjected to pharmacologically mediated diminished bone remodeling.

## Conclusions

Several medical societies have pointed to the problem of BP-associated jaw osteonecrosis, sought to clarify this phenomenon, and tried to educate treating physicians and patients [105], [106]. An important approach to reducing the incidence of BP-associated jaw osteonecrosis is to complete necessary oral surgery or larger jaw osteotomies prior to initiating this drug regimen [105], [106], [107], [108]. Increased public relations and educational work of specialist workers are needed for dental prophylaxis with planned BP medication and advice on the dental and surgical management of patients who have already received BP therapy [105], [106]. Several reviews have pointed to the fact that, at present, there appears to be no risk of OI patients suffering from BP-associated jaw osteonecrosis [53], [81], [82]. Our own experiences and the evaluation of the literature data also justify the conclusion that OI patients taking BP medication can be successfully treated for malposition of the teeth and malocclusion of jawbones, considering the difficult treatment conditions. In the published cases, bone healing was apparently unaffected by the medication, and the skeletal positioning remained stable. In this group of patients, the knowledge about the health status of the patient is also a key component of a customized and low-risk therapy.

## Notes

### Abbreviations

Nitinol is an acronym for a distinct metal alloy (“**Ni**ckel **Ti**tanium **N**aval **O**rdnance **L**aboratory”). 

### Informed consent

Informed consent has been obtained from the patient for publishing the case and using photographs of the patient.

### Competing interests

The authors declare that they have no competing interests.

### Conference presentation

The results of this study were presented in part on the occasion of the 94^th^ European Orthodontic Society (EOS) Congress, Edinburgh, Scotland, June 17–21, 2018. 

### Acknowledgements 

The authors appreciate the help of Shih-Jan Chin, DMD, Hamburg, for translating the article written in Japanese. The authors thank Prof. Zustin, pathologist, Hamburg, for the examination of the bone sample.

## Figures and Tables

**Table 1:**
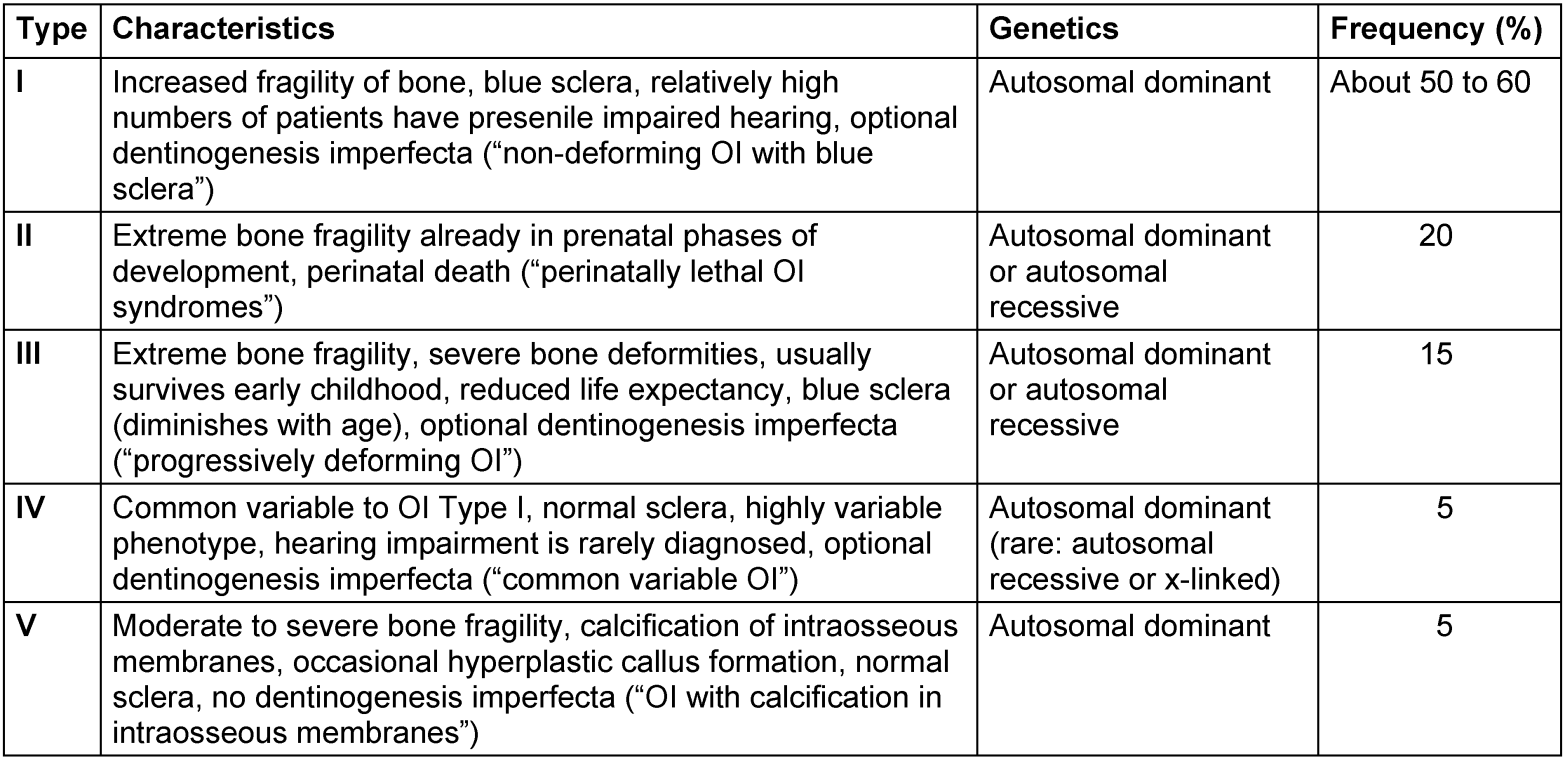
Classification of osteogenesis imperfecta (OI). The classification is based on the proposals and descriptions of Van Dijk and Sillence [4]. The optional presence of dentinogenesis imperfecta increases the likelihood of further skeletal changes and symptoms. The frequencies are approximations to the observed phenotypes derived from the literature and vary according to the preferred classification criteria. Some authors divide patients with OI into further types beyond the presented classification, which numerically follow the presented number series.

**Table 2:**
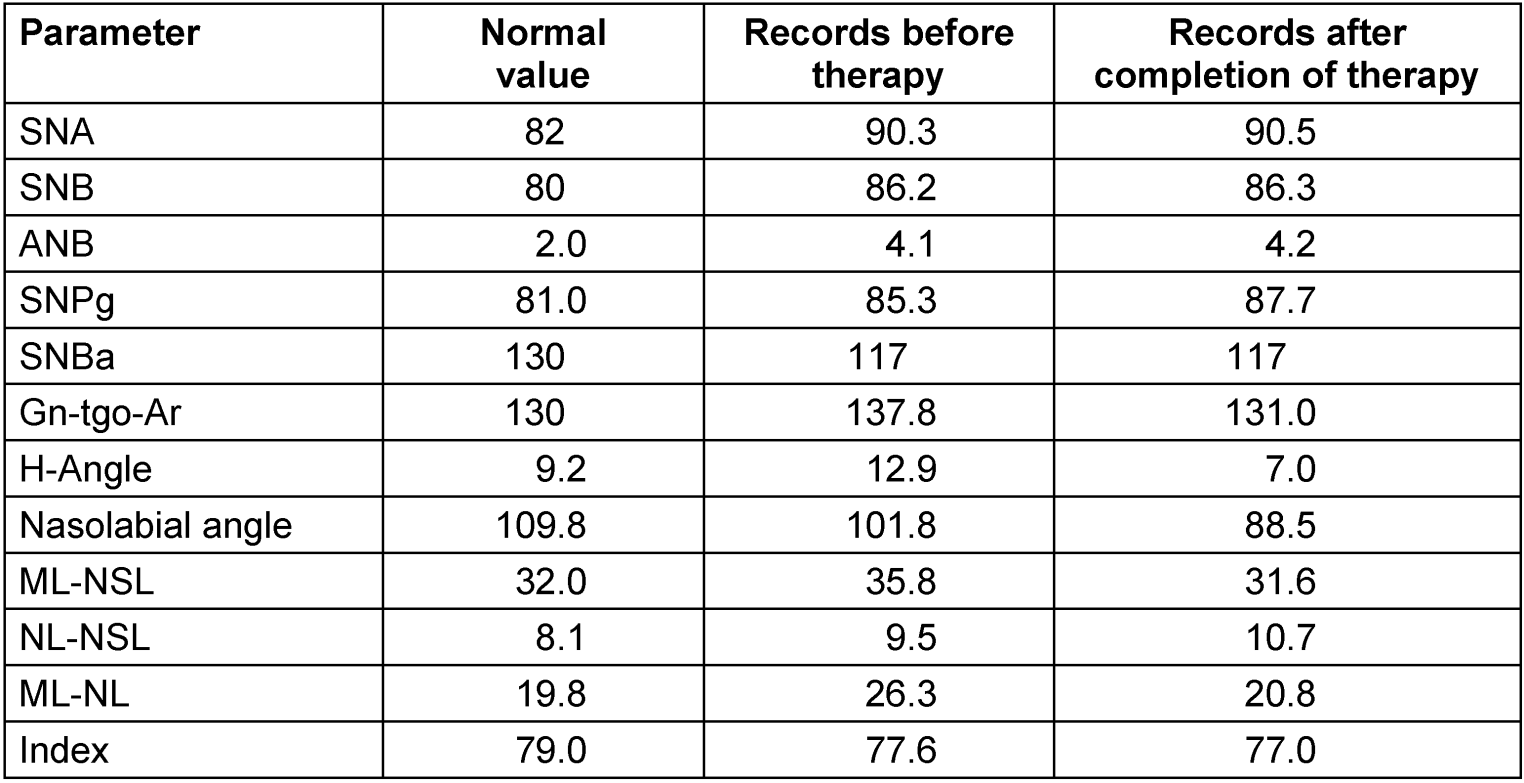
Cephalometric findings of the patient at the first examination and after completion of orthodontic-surgical therapy. Terms and abbreviations are defined in Table 3.

**Table 3:**
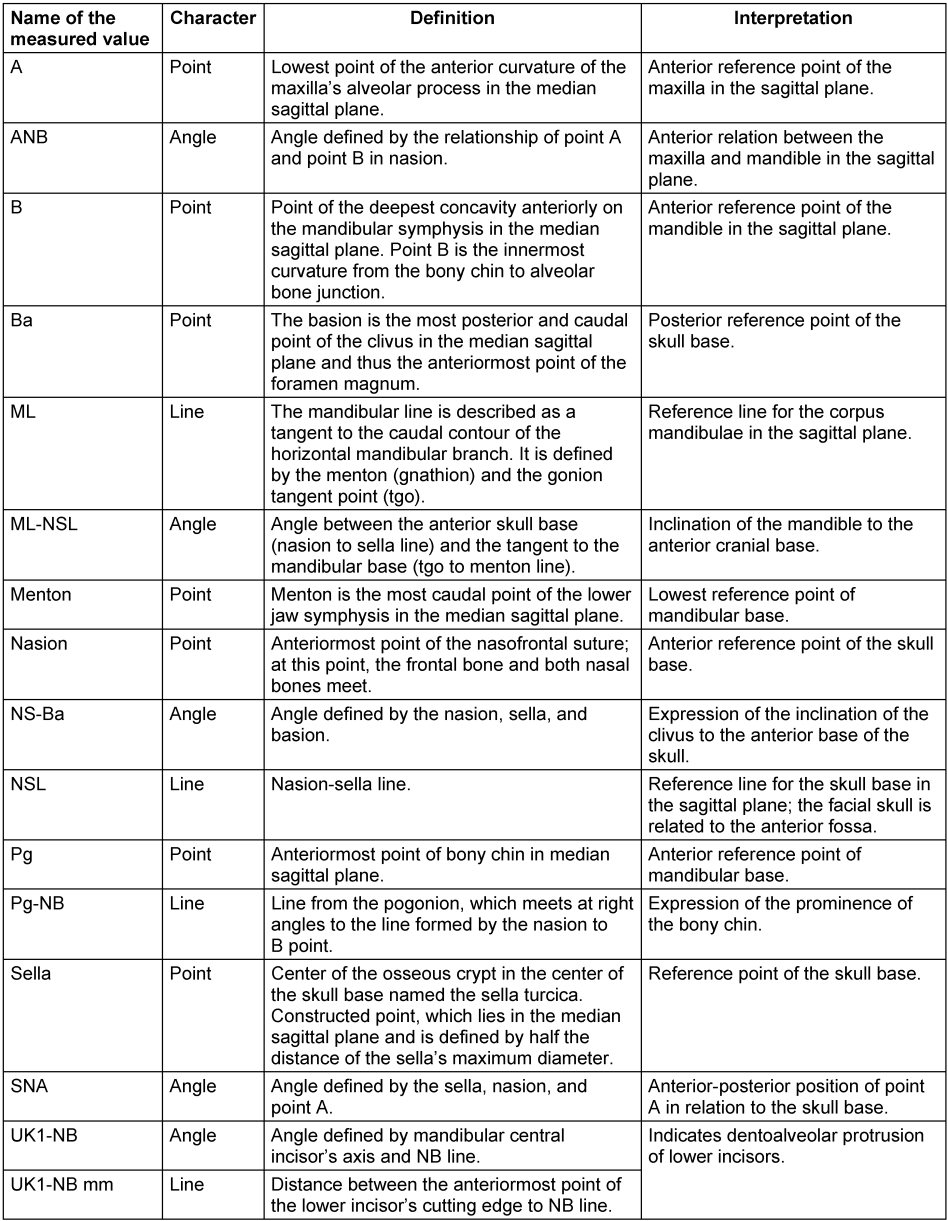
Definition of cephalometric terms

**Table 4:**
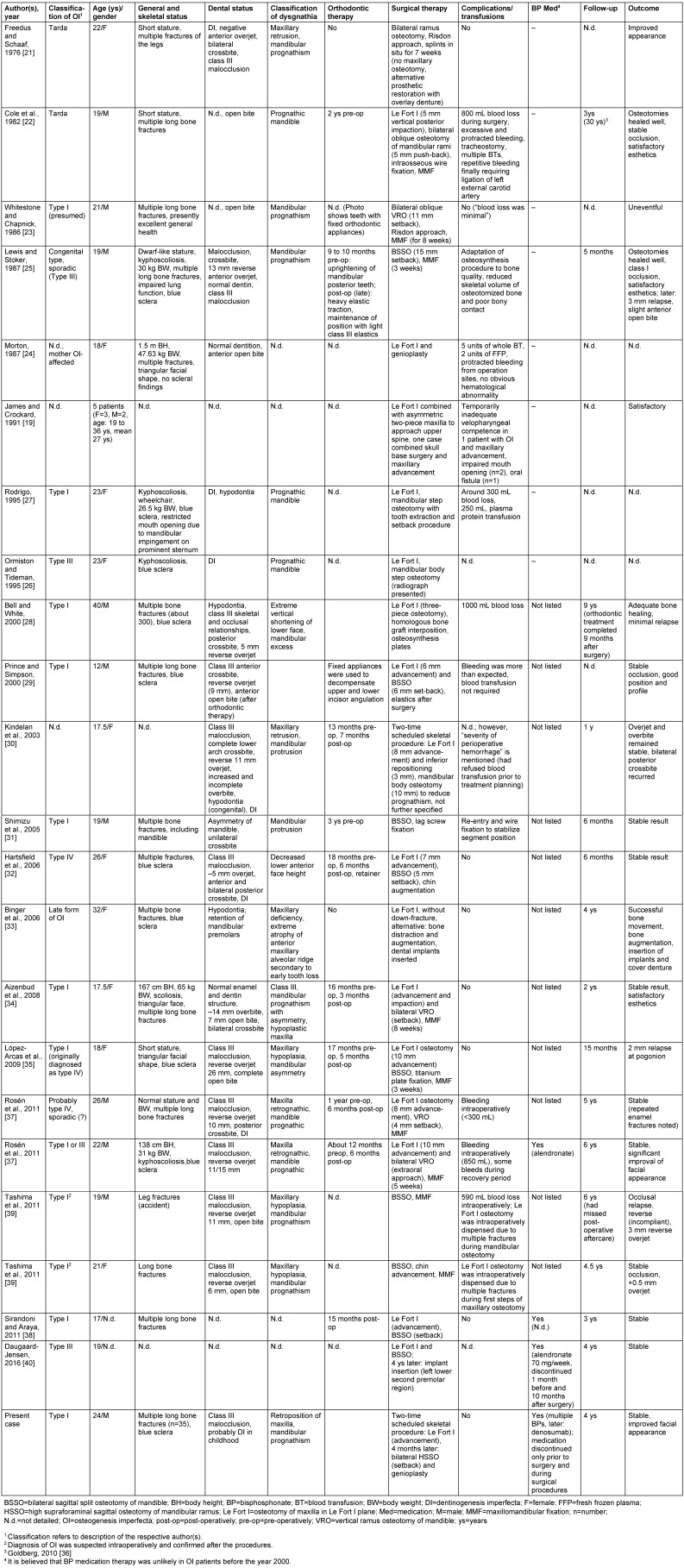
Literature review on orthognathic surgery in patients with osteogenesis imperfecta
